# Variants of the cry 1 gene may influence the effect of fat intake on resting metabolic rate in women with overweight of obesity: a cross-sectional study

**DOI:** 10.1186/s12902-021-00860-0

**Published:** 2021-10-05

**Authors:** Atieh Mirzababaei, Elnaz Daneshzad, Farideh Shiraseb, Sanaz Pourreza, Leila Setayesh, Cain C. T. Clark, Hadith Tangestani, Faezeh Abaj, Habib Yarizadeh, Khadijeh Mirzaei

**Affiliations:** 1 Department of Community Nutrition, School of Nutritional Sciences and Dietetics, Tehran University of Medical Sciences (TUMS), P.O.Box:14155-6117, Tehran, Iran; 2 Non-Communicable Diseases Research Center, Alborz University of Medical Sciences, Karaj, Iran; 3 Centre for Intelligent Healthcare, Coventry University, Coventry, CV1 5FB UK; 4 Department of Nutrition, Persian Gulf Tropical Medicine Research Center, Bushehr University of Medical Sciences, Bushehr, Iran

**Keywords:** Cry 1, Fat intake, SFA, PUFA, Resting metabolic rate, Obesity, Overweight, Interaction

## Abstract

**Background:**

Previous studies have shown that the minor allele (C allele) for Cry 1 rs2287161, may be associated with increased risk of cardiovascular diseases (CVDs). Low resting metabolic rate (RMR) caused by the diet has been shown to have, potentially, unfavorable effects on obesity. This study sought to investigate the interactions between the Cry 1 Gene and fat intake on RMR in women with overweight of obesity.

**Methods:**

This comparative cross-sectional study was conducted on 377 Iranian women with overweight of obesity. A food frequency questionnaire (FFQ), with 147 items, was used to assess dietary intake. Individuals were categorized into two groups based on the rs2287161 genotype. Body composition, dietary intake, and RMR were assessed for all participants.

**Results:**

There was a significant difference between genotypes for fasting blood sugar (FBS) (*P* = 0.04), fat free mass (FFM) (*P* = 0.0009), RMR per FFM (*P* = 0.05), RMR per body mass index (BMI) (*P* = 0.02), and RMR deviation (*P* = 0.01). Our findings also showed significant interactions between total fat and C allele carrier group on RMR per kg body weight, RMR per body surface area (BSA), RMR per FFM, and RMR deviation (P for interaction < 0.1), in addition to a significant interaction between CC + CG group genotype and polyunsaturated fatty acids (PUFA) intake on RMR per BMI (P for interaction =0.00) and RMR per kg (P for interaction = 0.02) and RMR per BSA (*P* = 0.07), compared to the GG group, after control for confounder factors.

**Conclusion:**

These results highlight that dietary compositions, gene variants, and their interaction, should be acutely considered in lower RMR.

## Introduction

The prevalence of overweight and obesity has increased such that almost one-third of the global population is now categorized as overweight or obese [1, 2]. Globally, obesity is almost 50% more prevalent among women [3, 4], primarily attributed due to a 3–5% lower resting metabolic rate (RMR) compared to men [5]. Obesity may be defined by an abnormal or excessive fat accumulation that leads to health impairment [6]. Moreover, obesity is associated with diabetes mellitus, cardiovascular diseases (CVDs) and some types of cancer [7–9], Previous studies propose the contribution of genetics, dietary, and environmental factors may play a significant role in the pathogenesis of obesity [10–13].

It has been theoretically demonstrated that individuals with low resting metabolic rate (RMR) are at increased risk of developing obesity-related disorders, since a larger portion of their daily food intake is stored as fat [14, 15]. RMR is affected by age, sex, body weight, pregnancy and hormonal status [16]. Indeed, RMR accounts for 60% of total energy expenditure (TEE) in individuals with sedentary habits [17], and it is highly determined by body composition, specifically fat-free mass (FFM) [18]. Energy intake and FFM are strongly linked [19], and, by extension, RMR is associated with energy intake [20]. Indeed, as adipose tissue increases in obese adults, fat mass (FM) poses a greater influence on RMR [21]. Different body composition indices, such as weight, lean body mass and body cell mass are inter-related [22], thus, various ratios of RMR are used among individuals.

The human circadian clock is responsible for the coordination between energy intake and metabolism based on changes in external factors including sunset/sunrise, physical activity, and dietary intake [23–25]. Recent findings show that regulation of metabolism by the circadian clock and its components is reciprocal. At the molecular level, the central circadian clock consists of Clock (circadian locomotor output cycles kaput), Bmal1 (brain and muscle Arnt like protein-1), Per (period)1,2,3, and Cry1,2 (cryptochrome) genes [26]. According to experimental studies, Cry1 plays a major role in lipid metabolism [27]. Indeed, hepatic depletion of CRY proteins increases circulating glucose, and their overexpression leads to a decrease in fasting blood glucose and improvement of insulin resistance in obese mice [28]. As part of circadian rhythmicity, these genes interact with the daily pattern of food intake [29]. It has previously been shown that Cry-deficient mice were more susceptible to obesity following a high-fat diet, than non-deficient counterparts [30]. Furthermore, a reduction of serum leptin due to any maladjustment of circadian rhythm and high fat diet-induced hyperinsulinemia, which stimulates lipogenesis, could alter energy homeostasis [30, 31]. It must be mentioned that circadian rhythm regulates metabolism via linking the Suprachiasmatic nucleus (SCN) to energetic c enters in the hypothalamus and brain stem. Conversely, metabolism regulates the circadian system; hormones that regulate metabolism can persuade or unset circadian rhythms [32, 33]; indeed, expression of Bmal1, Per2, and Cry1 in human subcutaneous and visceral fat [24] could lead to insulin resistance, inflammatory responses, reduced RMR, and higher body weight [34, 35].

Some studies have reported that RMR rate have depends on genetic factor and diet especially fat. Overall, some recent research, diet such as high-fat diet can have an interaction with CRY1 gene polymorphism [25, 36, 37]. Besides, previous studies show the CRY1 variant is associated with obesity and insulin resistance [38]. Although the exact mechanism underlying the association between RMR and IR has not been clear yet, recent data has indicated that a central pacemaker in the circadian system plays a role in controlling glucose homeostasis and energy metabolism basically along with each other [39]. Energy expenditure, which is tightly regulated by circadian rhythm, has a key role in obesity [40].

To our knowledge, there is currently no study that has investigated the association between Cry1, diet, and energy expenditure. Thus, given the potential future importance to clinical practice, we sought to assess the interaction of Cry1 and high-fat diet with RMR in women with overweight of obesity.

## Methods

### Study population

This cross-sectional study was conducted in 377 women, who were referred to health centers in Tehran, Iran from 2017 to 2019. Participants who had, self-certified, good general health were included in the study. The age of women ranged between 18 and 48 years, and their body mass index (BMI) ranged between 25 and 45 kg/m^2^. The exclusion criteria were; history of diabetes mellitus, hypertension, CVDs or fatty liver, taken all types of medicine including an oral contraceptive pill, smoking, intake of alcohol, pregnancy, currently lactating, and post menopause. We also excluded participants if chronic disease affected their diet, were following an arbitrary special dietary regimen, had weight fluctuations in the past 1 year, and if they were on a specific diet or if their daily energy intake was < 800 kcal or > 4200 kcal [41]. Anthropometrics, RMR measurements, biochemical markers, and DNA extraction were measured in the school of Nutritional Sciences and Dietetics at Tehran University of medical sciences (TUMS). Before commencing in this study, each participant signed a written informed consent form. Ethical approval, and associated number IR.TUMS.VCR.REC.1398.051 was obtained from the Ethics Commission of the TUMS.

### Assessment of high fat intake

A semi-quantitative, standard food frequency questionnaire (FFQ) was used to assess dietary intake, which was previously validated and adapted for this population [42]. The FFQ included 147 foods commonly consumed by Iranians, which were defined by standard serving sizes for each food item. FFQ data were collected through face-to-face interviews by trained interviewers at the health centers in Tehran. The software program, Nutritionist IV, was used for nutrient analysis, and was modified for Iranian foods [43].

To calculate fat intake, we first adjusted fat intake to energy, and then the associated percentage was calculated as the total daily caloric intake, where above 30% was defined as high fat intake and < 30% defined as low fat intake. Also, for saturated fatty acid (SFA) and poly-saturated fatty acid (PUFA), medians of SFA (Low < 25.76 g/d, High ≥25.76) and PUFA (Low < 18.8, High ≥18.81) intake were applied in statistical analysis.

### Anthropometric measurements

Weight was measured using a digital weighing scale, where participants wore light indoor clothing, were unshod, and recorded to the nearest 100 g. Height was measured to the nearest 0.5 cm while participants were in the normal standing position, without shoes, using a standard stadiometer (Seca, Germany). Waist circumference (WC) was measured at the umbilicus and recorded to the nearest 0.5 cm. A plastic tape measure was used to assess and hip circumferences (HC), to the nearest 0.5 cm, then, the ratio between waist and hip (WHR) circumferences was calculated. BMI was computed from the height and weight data, using the standard, weight (kg)/height^2^ (m^2^), equation.

### Resting metabolic rate (RMR) measurement

Resting metabolic rate was measured for all participants by a trained and experienced nutritionist using indirect calorimetry spirometer MetaLyzer 3B-R3 (Cortex. Biophysik GmbH, Leipzig, Germany). According to the manufacturer’s instructions, gas ventilation and exchange was calibrated before each test. High-resolution spiroergometric systems, with an infrared sensor, were used for CO_2_ evaluation and an amperometric solid electrolyte sensor for O_2_ evaluation, which were both recorded continuously through breath-by-breath gas analysis. Utilizing an ergonomically designed mask, a small portion of breathed air was conducted through the volume flow sensor. The RMR is evaluated by measuring the amount of O_2_ consumed and CO_2_ produced. Subjects were asked to avoid caffeine or alcohol consumption and vigorous exercise for a day and 12 h fasting before RMR measurements was required. The RMR was measured in the morning after a restful night’s sleep in a silent room with an ambient temperature of 24–26 °C. After achieving steady state in the supine position in a quiet and darkened atmosphere, the RMR was measured for 30 min. Gas exchange and ventilation were recorded continuously via breath-by-breath gas analysis. The oxygen uptake (VO2) and respiratory exchange ratio were analyzed within the last 20 min of the resting period and during a minimum of 5 consecutive minutes in steady-state conditions. Predictive RMR was determined using the Harris-Benedict equation, which considers the weight, height, and age of participants [44]. Participants were classified to two groups, low and high RMR, based on median values for; RMR per body surface area (BSA) (854.50), RMR deviation (− 8.00), RMR per BMI (50.90), and RMR per FFM (33.73), and 20 kcal/24 h /kg for RMR per kg body weight, according to the findings as reported previously in detail [45].

### Assessment of other variables

The International Physical Activity Questionnaire (IPAQ) was used to assess Physical Activity (PA), and was reported as metabolic equivalent hours per week (METs h/week) [46]. Activity levels were classified into low (< 600METs), moderate (600–3000 METs), and high (≥3000 METs) levels, according to the IPAQ scoring protocol. A demographic questionnaire (information on age, marital status, education, economic and job status) at study commencement.

### DNA extraction and sequencing of the gene

The Cry 1 gene primer was selected based on a previous study [47]. All participants from whom deoxyribonucleic acid (DNA) samples were accessible, were evaluated to be genotyped for the rs2287161. According to the manufacturer’s protocol, we extracted genomic DNA from blood samples with the use of the Mini Columns, Type G kit (GeneALL, Exgene) The concentration and quality of the extracted DNA were assessed by the use of a Nano Drop ND-2000 spectrometer. The rs2287161 (minor allele: C; major allele: G) was genotyped by polymerase chain reaction-restricted length polymorphism (PCR–RFLP) technique. PCR applied the following primers: forward 5′-GGAACAGTGATTGGCTCTATCT − 3′; reverse 5′-GGTCCTCGGTCTCAAGAAG-3′. PCR reactions were performed in a final volume of 20 μl include of 2 μl primers, 1 μl extracted DNA,7 μl distilled water, and 10 μl Taq DNA Polymerase Master Mix (Amplicon; Denmark) with the next conditions in a DNA thermocycler: The DNA templates were denatured at 94 °C for 4 min; amplification contained of 35 cycles at 94 °C, 58 °C and 72 °C (each stage for 30 s), with a final extension at 72 °C for 7 min. Amplified DNA (10 μl) was mixed with 2 μl of DRI restriction enzyme (Thermo Fisher Scientific; USA) at 37 °C. To ensure the PCR process and amplification of the desired parts, PCR products electrophoresis was performed on agarose gel. Fragments including three possible genotypes were then determined: uncut homozygous GG (107 bp), cut heterozygous GC (107,48 and 226 bp), and cut homozygous CC (155 and 226 bp). In order to examine the interactions between fat intake, SFA, PUFA intake, and Cry 1 polymorphisms on RMR, the participants were grouped based on Cry 1 polymorphisms: group 1 with GG (rs2287161) genotype (*n* = 107), group 2 or C allele carrier group with CC and GC genotype (*n* = 270).

### Laboratory tests

All samples were collected, after 10–12 h fasting, at the laboratory of the school of Nutritional and Dietetics at TUMS. Fasting serum glucose, insulin, total cholesterol, triglyceride (TG), low density lipoprotein (LDL), and high density lipoprotein (HDL) were measured from blood samples. IR was calculated by the homeostatic model assessment (HOMA) according to the following equation: HOMA-IR = [fastingplasma glucose (mmol/l) * fasting plasma insulin (mIU/l)]/22.5.

### Statistical analysis

The Hardy-Weinberg equilibrium and comparison of categorical variables were assessed with the χ2 test. Descriptive statistics, including the mean (standard deviation) and frequency summaries, were used to describe the study population. A total of 377 Iranian women with overweight of obesity were categorized based on rs2287161genotypes and divided into two groups according to dominant genetic model (risk allele carriers CG + GC genotype (*n* = 270) versus homozygous non-risk allele GG genotype (*n* = 107). Comparisons between groups were made using the independent t-test for continuous variables and chi-square test for categorical variables. Moreover, age, BMI, IPAQ, and energy intake-adjusted analyses were performed using general linear models (ANCOVA). Moreover, to analyze the potential interactions between genotype and diet intake, and the genotype and fat, PUFA or SFA, an interaction term of genotype x fat, SFA or PUFA dietary intake on types of RMR was included in the binary logestic regression. For this analysis, the GG genotype and categories of lower intake of total fat, PUFA, SFA were considered as reference groups. Data were analyzed using IBM SPSS version 23 (SPSS, Chicago, IL, USA). *P*-values for all variables were reported before the adjustment in the crude model by by independent T-test and after adjustment with potential confounders as age, BMI, physical activity, and energy intake using analysis of covariance (ANCOVA). *P* < 0.05 was considered statistically significant, but for interactions, *P* < 0.1 was considered significant.

## Results

### Study population characteristics

The present study was conducted on 377 obese and overweight Iranian women, of which, 70.8% were married, 36.2% occupied, 86.6% had a college education, and 45.8% had good economic status. The mean age, weight, BMI, WHR, WC, body fat mass (BFM), FFM were 36.67 ± 9.10 years, 81.29 ± 12.43 kg, 31.26 ± 4.29 kg/m^2^, 1.16 ± 4.54, 99.61 ± 10.07 cm, 34.74 ± 8.75 kg, 46.52 ± 5.71 kg, respectively. The mean of RMR in the study population was 1574.96 ± 259.71. The median of RMR groups for binary analysis was considered for analysis as following RMR per BSA (854.50), RMR deviation (− 8.00), RMR per BMI (50.90), and RMR per FFM (33.73), and for RMR per kg body weight was [20], respectively. Also, the mean intake of total dietary fat intake was 95.13 ± 35.17 g, SFA 28.40 ± 7.43 g, and PUFA 20.08 ± 7.57 g, respectively. The overall prevalence of rs2287161 genotypes in participants for CC+ CG and GG was 66.8 and 26.5%, respectively.

### Study participant characteristics between genotype of rs2287161

Comparison of participant’s variables based on rs2287161 genotypes was shown in Table 1. After genotype classification, we found significant differences in the crude model among genotypes for age (*P* = 0.03), FFM (*P* = 0.00), BMI (*P* = 0.06), RMR per BMI (*P* = 0.02), RMR per FFM (*P* = 0.05) RMR deviation (*P* = 0.01), FBS (*P* = 0.04), marriage status (*P* = 0.07), economic status (*P* = 0.01), and physical activity (*P =* 0.04).

**Table 1:** Characteristics of study population according to rs2287161 genotypes

Variables	rs2287161 genotypes			
	***CC + CG*** (*n* = 270 (	***GG*** (*n* = 107)	p-value*	p-value**
**Age (year)**	37.31 ± 9.40	35.03 ± 8.30	**0.03**	**0.07**
**Body composition**				
**Weight (kg)**	81.29 ± 12.31	80.01 ± 11.57	0.35	0.80
**Height (cm)**	160.88 ± 5.73	161.81 ± 5.66	0.15	0.72
**FFM (kg)**	46.36 ± 5.64	46.46 ± 5.58	**0.00**	0.70
**BMI (kg/m**^**2**^**)**	31.40 ± 4.24	30.53 ± 4.04	**0.06**	0.87
**BFM (kg)**	35.01 ± 8.72	33.48 ± 7.88	0.11	0.77
**WHR**	0.93 ± 0.05	0.93 ± 0.05	0.41	0.43
**WC (cm)**	99.82 ± 9.99	98.30 ± 9.39	0.17	0.52
**RMR measurement**				
**RMR** (**kcal/day)**	1568.58 ± 247.09	1586.30 ± 278.82	0.59	0.77
**RQ**	0.85 ± 0.043	0.85 ± 0.03	0.76	0.82
**RMR per Kg body weight** **(kcal/day/kg)**	19.64 ± 3.06	19.87 ± 3.16	0.55	0.98
**RMR per BSA** **(kcal/day/m**^**2**^**)**	850.57 ± 106.57	857.47 ± 127.64	0.64	0.83
**RMR per BMI** **(kcal/day/kg/m**^**2**^**)**	51.14 ± 8.09	52.38 ± 9.71	**0.02**	0.86
**RMR per FFM** **(kcal/day/kg)**	33.76 ± 4.14	34.14 ± 4.99	**0.05**	0.97
**RMR deviation** (%)	−8.38 ± 11.89	−7.79 ± 13.91	**0.01**	0.99
**Biochemical assessment**				
**FBS (mg/dL)**	88.39 ± 10.30	85.71 ± 7.97	**0.04**	0.11
**HOMA-IR (mg/dL)**	3.38 ± 1.30	3.36 ± 1.26	0.91	0.56
**TC (mg/dL)**	184.07 ± 34.45	187.22 ± 38.26	0.52	**0.09**
**HDL (mg/dL)**	46.57 ± 11.58	46.16 ± 9.92	0.78	0.46
**LDL (mg/dL)**	94.80 ± 24.39	95.14 ± 23.94	0.91	0.83
**TG (mg/dL)**	118.89 ± 59.06	118.39 ± 60.44	0.95	0.88
**hs CRP (mg/L)**	4.30 ± 4.80	3.93 ± 3.90	0.57	0.66
**IPAQ**				
**Low**	82 (70.7%)	39 (29.3%)	**0.04**	0.98
**Moderate**	100 (65.4%)	40 (34.6%)		
**High**	41(35.4%)	75 (64.6%)		
**History of weight loss in past years**				
**Yes**	86 (31.8%)	122 (68.2%)	**0.08**	0.13
**No**	49 (24.5%)	120 (75.5%)		

Quantitative variables were reported with mean and SD and qualitative variables with number and percentage

*P values resulted from the independent T.test for continuous variables and chi-square test for categorical variables

**P-value is found by ANCOVA and adjusted for age, BMI, physical activity, and total energy intake

*BMI* body mass index, *WC* waist circumference, *WHR* waist-to-hip ratio, *FFM* fat free mass, *HDL* high density lipoprotein, *hs-CRP* high-sensitivity C reactive protein, *LDL* low density lipoprotein, *BMR* basal metabolic rate, *TG* triacylglycerol, *TC* total cholesterol, *PUFA* poly unsaturated fatty acid, *SAFA* saturated fatty acid, *HOMA* homeostatic model assessment, *GLU* Glucose, *RMR* resting metabolic rate, *RQ* respiratory quotient, *RMR/BSA* resting metabolic rate per body surface area, *RMR/FFM* resting metabolic rate per fat free mass, *RMR/BMI* resting metabolic rate per body mass index

Cut point IPAC: low < 600 METs, moderate:600–3000 METs, high> 3000 METs

Also, after controlling for confounders, age remained marginally significant (*P =* 0.07) with a higher mean in the group with risk allele group (CC + CG), and in education status (*P =* 0.01). For all other variables, no significant association was observed (Table 1).

### Association between general characteristics of participants in three grouped of SFA (gr/d), PUFA (gr/d), and fat intake (gr/d) among the population

General characteristics of participants, such as body composition, biochemical assessment, RMR measurement, and others among lower vs. higher than the median of total fat, trans fatty acid (TFA), and polyunsaturated fatty acid (PUFA) intake, are presented in Table 2.

**Table 2:** General characteristics of participants in three grouped of SFA (gr/d), PUFA (gr/d), and fat intake (gr/d) among studied population

Variables	SFA intake (gr/d)				PUFA intake (gr/d)				Total Fat Intake(%)			
	***Low*** ***< 25.76***	***High*** ***≥25.76***	p-value*	p-value**	***Low*** ***< 18.81***	***High*** ***≥18.81***	p-value*	p-value**	***Low*** ***< 30%***	***High*** ***≥30%***	p-value*	p-value**
**Age (year)**	37.24 ± 9.15	36.42 ± 9.23	0.40	0.27	37.29 ± 9.19	36.10 ± 9.21	0.20	0.15	35.46 ± 9.07	37.32 ± 9.07	**0.05**	0.78
**Body composition**												
**Weight (kg)**	81.38 ± 10.99	81.06 ± 12.86	0.80	0.97	81.17 ± 12.02	81.16 ± 12.52	0.99	0.85	82.61 ± 13.11	80.59 ± 12.02	0.12	0.59
**Height (cm)**	161.00 ± 5.70	161.222 ± 5.98	0.73	0.48	161.52 ± 6.06	160.77 ± 5.69	0.20	0.64	162.13 ± 5.58	160.74 ± 5.96	**0.02**	0.76
**FFM (kg)**	47.02 ± 5.68	46.23 ± 5.65	0.19	0.52	46.84 ± 5.57	46.15 ± 5.75	0.22	0.54	47.48 ± 5.75	46.01 ± 5.63	0.10	0.95
**BMI (kg/m**^**2**^**)**	31.46 ± 3.93	31.18 ± 4.47	0.24	0.60	31.14 ± 4.31	31.40 ± 4.30	0.56	0.73	31.45 ± 4.52	31.45 ± 4.17	0.50	0.72
**BFM (kg)**	34.62 ± 7.52	34.78 ± 9.30	**0.04**	0.99	34.34 ± 8.58	35.12 ± 8.90	0.32	0.39	35.25 ± 9.43	34.46 ± 8.37	0.38	0.35
**WHR**	0.93 ± 0.04	1.28 ± 5.64	0.47	0.29	1.40 ± 6.52	0.93 ± 0.05	0.05	0.90	0.93 ± 0.05	1.28 ± 5.63	0.46	0.14
**WC (cm)**	99.68 ± 8.86	99.54 ± 10.64	**0.02**	0.90	99.73 ± 9.73	99.44 ± 10.42	0.77	0.91	100.37 ± 10.36	99.20 ± 9.91	0.26	0.39
**Biochemical assessment**												
**FBS (mg/dL)**	86.98 ± 9.26	87.75 ± 9.82	0.55	0.53	87.18 ± 9.67	87.83 ± 9.60	0.59	0.67	86.37 ± 8.06	77.08 ± 10.36	0.18	0.11
**HOMA-IR (mg/dL)**	3.28 ± 1.20	3.46 ± 1.45	0.33	**0.02**	3.34 ± 1.20	3.32 ± 1.35	0.89	0.73	3.31 ± 1.04	3.37 ± 1.37	0.74	0.27
**TC (mg/dL)**	182.49 ± 32.27	186.50 ± 38.14	0.41	0.28	189.47 ± 38.12	180.53 ± 33.69	**0.05**	0.10	184.64 ± 37.14	186.54 ± 33.16	0.68	**0.06**
**HDL (mg/dL)**	45.06 ± 10.45	47.69 ± 10.96	**0.07**	0.12	45.77 ± 11.45	47.90 ± 9.99	0.12	0.23	46.76 ± 11.90	46.48 ± 10.31	0.84	0.48
**LDL (mg/dL)**	90.75 ± 21.53	97.20 ± 25.22	**0.01**	**0.02**	95.51 ± 24.51	94.51 ± 23.93	0.74	0.46	94.67 ± 22.50	95.63 ± 24.99	0.76	0.38
**TG (mg/dL)**	132.74 ± 74.84	112.98 ± 51.67	**< 0.001**	0.87	122.15 ± 59.45	115.14 ± 60.32	0.36	0.95	128.24 ± 66.37	113.23 ± 54.47	**0.05**	0.63
**hs. CRP (mg/L)**	3.70 ± 4.01	4.62 ± 4.92	0.15	**0.08**	3.94 ± 4.51	4.69 ± 4.77	0.22	0.63	4.39 ± 4.42	4.32 ± 4.74	0.91	0.81
**RMR measurement**												
**RMR** (**kcal/day)**	1565.08 ± 241.12	1582.93 ± 268.67	0.58	0.30	1566.88 ± 259.51	1586.74 ± 259.88	0.51	0.39	1590.78 ± 256.01	1566.89 ± 261.85	0.45	0.16
**RQ**	0.85 ± 0.04	0.85 ± 0.04	0.75	0.88	0.85 ± 0.04	0.85 ± 0.04	0.66	0.27	0.85 ± 0.0	0.85 ± 0.04	0.79	0.75
**RMR per Kg body weight** **(kcal/day/kg)**	19.38 ± 3.16	19.79 ± 3.09	0.30	0.15	19.57 ± 3.17	19.73 ± 3.07	0.67	0.26	19.53 ± 3.24	19.62 ± 3.03	0.81	0.33
**RMR per BSA** **(kcal/day/m**^**2**^**)**	846.30 ± 116.32	855.46 ± 116.32	0.52	0.31	847.98 ± 115.27	856.68 ± 114.02	0.52	0.35	853.25 ± 111.15	848.66 ± 115.51	0.80	0.20
**RMR per BMI** **(kcal/day/kg/m**^**2**^**)**	50.80 ± 9.00	51.66 ± 8.76	0.43	0.24	51.34 ± 8.93	51.41 ± 8.77	0.94	0.43	51.68 ± 8.79	51.08 ± 8.74	0.90	0.18
**RMR per FFM** **(kcal/day/kg)**	33.28 ± 4.31	34.13 ± 4.57	0.12	0.11	33.26 ± 4.13	34.42 ± 4.77	**0.02**	**0.05**	33.57 ± 4.33	33.86 ± 4.55	0.46	**0.06**
**RMR deviation** **(%)**	−9.26 ± 12.94	−7.74 ± 12.56	0.34	0.22	−8.56 ± 13.18	−7.95 ± 12.22	0.69	0.39	−8.44 ± 12.59	−8.49 ± 12.44	0.97	0.28
**IPAQ**												
**Low**	40 (32.0%)	85 (68.0%)	0.15	0.39	64 (51.2%)	61 (48.8%)	0.80	0.87	40 (31.5%)	87 (68.5%)	0.7	0.51
**Moderate**	36 (31.3%)	79 (68.7%)			54 (47%)	64 (53%)			41 (34.5%)	78 (65.5%)		
**High**	7 (58.3%)	5 (41.7%)			6 (50%)	6 (50%)			3 (41.7%)	7 (58.3%)		
**rs2287161 genotypes**												
**CC + GC**	88 (33.3%)	176 (66.7%)	0.46	0.93	129 (48.9%)	135 (51.1%)	0.48	0.79	93 (34.4%)	177 (65.5%)	0.63	0.94
**GG**	37 (38.6%)	63 (62.4%)			54 (53.5%)	47 (46.5%)			40 (37.4)	67 (62.6%)		
**History of weight loss in past years**												
**Yes**	69 (35.4%)	126 (64.6%)	0.18	0.56	95 (48.7%)	100 (51.3%)	0.67	0.32	70 (35.7%)	126 (64.3%)	0.72	0.68
**No**	47 (28.8%)	116 (71.2%)			83 (50.9%)	80 (49.1%)			63 (37.5%)	105 (62.5%)		

Quantitative variables were reported with mean and SD and qualitative variables with number and percentage

values were calculated by Independent T.test as Mean ± SD

*P values resulted from the analysis of Independent T.test for continuous variables and chi-square test for categorical variables. We also performed a Tukey test to compare each genotype with other types for continuous variables

**P-value is found by ANCOVA and adjusted for age, BMI, physical activity, and total energy intake

*BMI* body mass index, *WC* waist circumference, *WHR* waist-to-hip ratio, *FFM* fat free mass, *HDL* high density lipoprotein, *hs-CRP* high-sensitivity C reactive protein, *LDL* low density lipoprotein, *BMR* basal metabolic rate, *TG* triacylglycerol, *TC* total cholesterol, *PUFA* poly unsaturated fatty acid, *SFA* saturated fatty acid, *HOMA* homeostatic model assessment, *GLU* Glucose, *RMR* resting metabolic rate, *RQ* respiratory quotient, *RMR/BSA* resting metabolic rate per body surface area, *RMR/FFM* resting metabolic rate per fat free mass, *RMR/BMI* resting metabolic rate per body mass index

Cut point IPAC: low < 600 METs, moderate:600–3000 METs, high> 3000 METs

#### General characteristics of participants among SFA intake categories

In the crude model, in body composition variables there were significant mean differences for BFM (*P* = 0.04), WC (*P* = 0.02), and in biochemical variables; TG (*P =* < 0.001). Among SFA categories, there was a significant mean difference for marriage status (*P =* 0.02). After adjusting for potential confounders, women with higher intake of SFA had significantly higher mean HOMA-IR (*P =* 0.02), and LDL(*P =* 0.02), all other variables were no longer significant after adjustment. Regarding other variables related to general characteristics, there were no significant differences noted (all *P* > 0.05).

#### General characteristics of participants among PUFA intake categories

There was a significant difference in cholesterol between lower and higher PUFA intake categories before adjustment (*P* = 0.05), but after controlling for confounders, this association was not present. There were no significant differences in terms of other biochemical assessments, body composition, RMR measurement, education level, economic status, marital status, rs2287161 genotypes, physical activity, and job-status (all *P* > 0.05) (Table 2).

#### General characteristics of participants among total fat intake category

There were significant differences in age (*P* = 0.05), TG (*P =* 0.05), height (*P* = 0.02), and marriage status (*P =* 0.02) between lower and higher total fat intake categories in the crude model, but after controlling for confounders (age, BMI, physical activity and total energy intake), these variables were no longer significant(*P* > 0.05). There were no significant differences for the remaining variables before and after adjustment (P > 0.05) (Table 2).

### Dietary intake of study population according to rs2287161 genotypes

The dietary intake of the participants across two groups of risk allele genotype as GG and GC + CG are shown in Table 3.

**Table 3:** Dietary intake of study population according to rs2287161 genotypes

rs2287161 genotypes	***CC + GC*** (*n* = 270) Mean ± SD	***GG*** (*n* = 107) Mean ± SD	***P value***	P value*
**Macronutrient**				
Energy (kcal)	2635.5 ± 798.17	2739.85 ± 827.69	0.27	–
Protein (gr)	91.98 ± 31.55	93.83 ± 32.08	0.61	0.44
Carbohydrate (gr)	372.11 ± 11.76	392.12 ± 130.94	0.17	0.91
Total fat (gr)	97.63 ± 33.70	95.21 ± 31.31	0.54	0.53
**Micronutrient**				
Trans.fat (gr)	0.0006 ± 0.001	0.0008 ± 0.001	0.87	0.48
Cholesterol (gr)	236.64 ± 111.65	272.52 ± 123.51	0.51	0.93
SAFA (gr)	28.84 ± 11.92	28.15 ± 10.72	0.61	**0.03**
MUFA (gr)	32.02 ± 12.42	32.74 ± 12.12	0.62	0.60
PUFA (gr)	19.93 ± 8.80	20.70 ± 9.09	0.45	0.94
Oleic (gr)	28.80 ± 11.59	29.37 ± 11.45	0.67	0.58
Linoleic (gr)	17.27 ± 8.21	17.93 ± 8.68	0.49	0.97
Linolenic (gr)	1.20 ± 0.62	1.20 ± 0.59	0.98	0.50
EPA (gr)	0.02 ± 0.03	0.03 ± 0.04	0.35	0.41
DHA (gr)	0.09 ± 0.11	0.10 ± 0.12	0.38	0.45
Total fiber(g)	47.30 ± 21.40	50.18 ± 21.64	0.25	0.56

Variables is presented by mean ± SD

*P* values resulted from the analysis of Independent T.test

*P*-value* is obtained by ANCOVA after adjustment for calories intake

*PUFA* poly unsaturated fatty acid, *SAFA* saturated fatty acid, *MUFA* mono saturated fatty acid, *EPA* Eicosapentaenoic acid, *DHA* docosahexaenoic acid

SFA intake was significantly lower in the GG genotype group compared to the CC + CG group (28.15 vs 28.84 g/day, *P* = 0.03). Table 3.

### The interactions between the intake of total fat, SFA, and PUFA intake, and rs2287161 genotypes on the different type of RMR

#### Interaction between different types of RMRs across total fat intake category

**Fig. 1:**
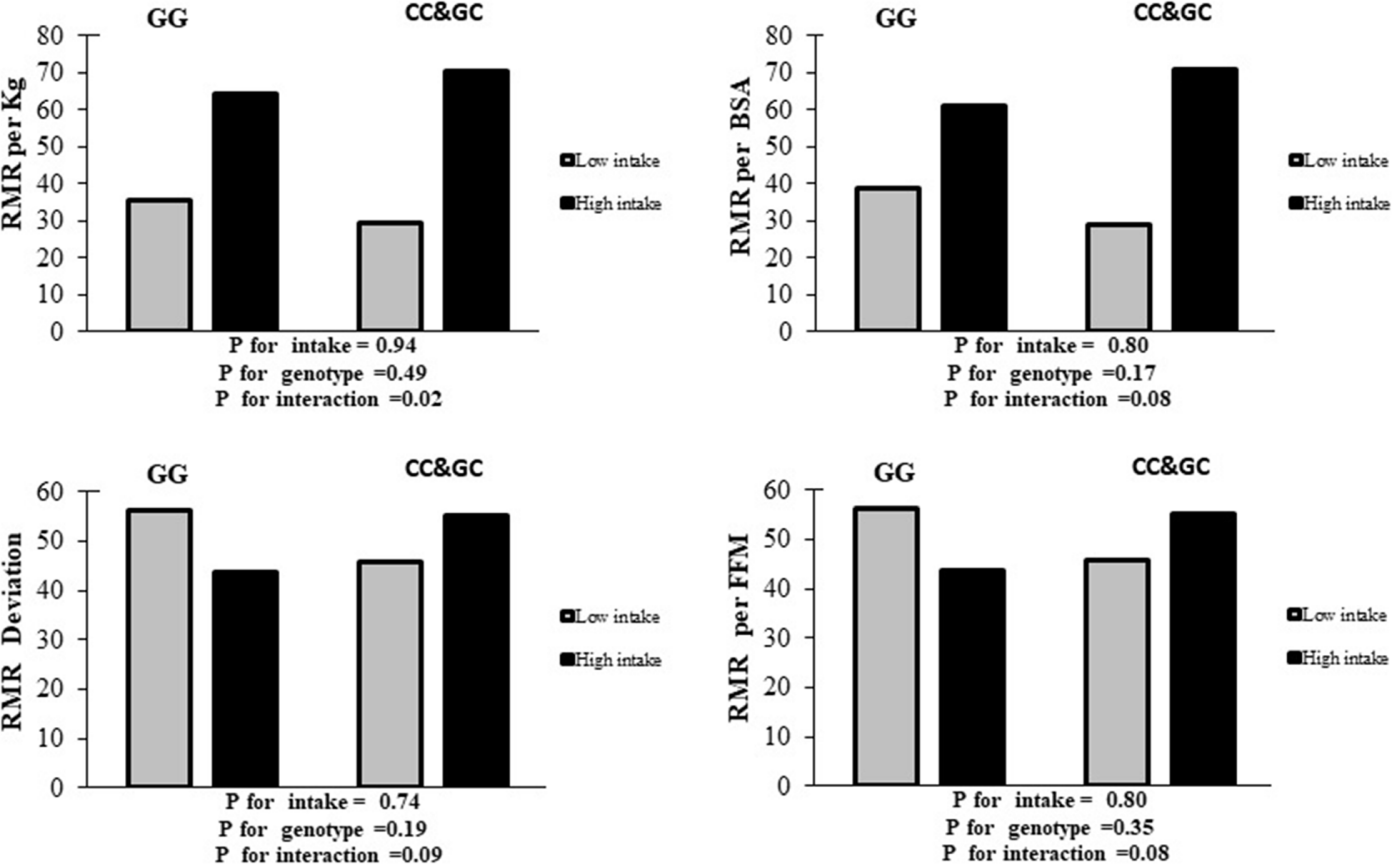
Interaction between dietary fat and Cry 1 genotypes on RMR disorder. Percentage of Types of RMR disorder across GC&CC and CC genotypes base on low and high dietary fat (% energy). A) Percentage of RMR per kg disorder in low intake across GG and GC&CC genotypes were respiratory 35.5 and 29.5%, Percentage of RMR/kg disorder in high intake across GG and GC&CC genotypes were respiratory 64.5 and 70.5%. B) Percentage of RMR per BSA disorder in low intake across GG and GC&CC genotypes were respiratory 38.8 and 28.9%., Percentage of RMR per BSA disorder in high intake across GG and GC&CC genotypes were respiratory 61.2%and 71.1. C) Percentage of RMR Deviation disorder in low intake across GG and GC&CC genotypes were respiratory 36.6 and 27.8%., Percentage of RMR Deviation disorder in high intake across GG and GC&CC genotypes were respiratory 63.4 and 72.2%. D) Percentage of RMR per FFM disorder in low intake across GG and GC&CC genotypes were respiratory 37.2 and 30.8%., Percentage of RMR per FFM disorder in high intake across GG and GC&CC genotypes were respiratory 62.8and 69.2%. *P for interaction is for model adjusted (Potential confounders: education level, BMI, marriage status, age, history of weight loss in past year, energy intake, economic status, RQ, and physical activity)

In the crude models, there was no significant interaction between CC + CG group genotypes and high fat intake on odds of RMR per kg body weight compared to the GG group (β:-0.65, OR:0.51; 95% CI:0.19–1.35, *P* = 0.18) but in Model 1, after adjusting for potential confounders, such as education level, BMI, marriage status, age, history of weight loss in past year, total energy intake, economic status, respiratory quotient (RQ), and physical activity, the association changed to a significant interaction (β:-1.55, OR: 0.21, 95%CI: 0.04–0.98, *P* = 0.02). The RMR per BSA variable in the crude model did not yield a significant interaction (β: -0.97, OR: 0.55, 95%CI: 0.13–1.18, *P* = 0.28), yet, after controlling for confounders, a significant interaction was found (β:-1.49, OR: 0.28, 95%CI: 0.05–0.92, *P* = 0.08). In addition, RMR per FFM was not significant in the crude model (β: -0.59, OR: 0.55, 95%CI: 0.18–1.64, *P =* 0.28), but, in the adjusted model, a significant interaction was found (β: -1.24, OR: 0.28, 95%CI:0.07–1.16, *P =* 0.08). Moreover, in the crude model, there was no significant interaction between the allele risk group (CC + CG) in comparison with the reference group (GG) on RMR deviation from normal (β:-0.77, OR: 0.46, 95%CI: 0.15–1.39, *P* = 0.17), however, after controlling for confounders, a significant interaction was found (β:-1.19, OR: 0.30, 95%CI: 0.07–1.24, *P* = 0.09) (Table 4, Fig. 1). No significant interaction was found between RMR per BMI and total fat intake (Table 4).

**Table 4:** Investigation of the interactions between intake of Fat, SAFA, and PUFA intake and rs2287161 genotypes on the different type of RMR

Variables	Models	Allele	High fat intake				PUFA intake				SAFA intake			
			β ± SE	95% CI	OR	P	β ± SE	95%CI	OR	P	β ± SE	95%CI	OR	P
**RMR per kg body weight** **(kcal/day/kg)**	**Crude**	GG	Reference											
		CG + CC	−0.65 **±** 0.49	0.19–1.35	0.51	0.18	−0.96 ± 0.48	0.14–0.97	0.38	**0.04**	−1.02 ± 0.51	0.13**–**0.97	0.35	**0.04**
	**Adjusted**	GG	Reference				Reference				Reference			
		CG + CC	− 1.55 ± 0.78	0.04–0.98	0.21	**0.02**	− 1.65 ± 0.74	0.04–0.82	0.19	**0.02**	− 1.01 ± 0.77	0.08–1.63	0.36	0.18
**RMR per BSA** **(kcal/day/m**^**2**^**)**	**Crude**	GG	Reference				Reference				Reference			
		CG + CC	−0.97 ± 0.56	0.13–1.18	0.55	0.28	− 0.94 ± 0.54	0.13–1.13	0.75	0.60	−0.51 ± 0.56	0.19–1.81	0.50	0.23
	**Adjusted**	GG	Reference				Reference				Reference			
		CG + CC	−1.49 **±** 0.72	0.05–0.92	0.28	**0.08**	− 1.22 ± 0.68	0.07–1.12	0.29	**0.07**	−0.45 ± 0.71	0.15–2.57	0.81	0.77
**RMR per BMI** **(kcal/day/kg/m**^**2**^**)**	**Crude**	GG	Reference											
		CG + CC	−0.77 **±** 0.55	0.15–1.38	0.46	0.16	−1.38 ± 0.55	0.08–0.73	0.25	**0.01**	−0.59 ± 0.56	0.18–1.67	0.55	0.29
	**Adjusted**	GG	Reference				Reference				Reference			
		CG + CC	−1.09 ± 0.77	0.07–1.52	0.33	0.15	− 1.97 ± 0.75	0.03–0.61	0.13	**0.009**	−0.63 ± 0.76	0.11–2.35	0.52	0.40
**RMR per FFM** **(kcal/day/kg)**	**Crude**	GG	Reference											
		CG + CC	−0.59 **±** 0.55	0.18–1.64	0.55	0.28	− 0.27 ± 0.54	0.25–2.20	0.75	0.60	−0.67 ± 0.25	0.16–1.54	0.50	0.23
	**Adjusted**	GG	Reference				Reference				Reference			
		CG + CC	−1.24 ± 0.71	0.07–1.16	0.28	**0.08**	−0.27 ± 0.67	0.20–2.83	0.76	0.68	−0.20 ± 0.71	0.20–3.34	0.81	0.77
**RMR Deviation** **(%)**	**Crude**	GG	Reference				Reference				Reference			
		CG + CC	−0.77 **±** 0.56	0.15–1.39	0.46	0.17	− 0.90 ± 0.54	0.13 to 1.18	0.40	**0.09**	− 0.23 ± 0.56	0.25–2.4	0.79	0.67
	**Adjusted**	GG	Reference				Reference				Reference			
		CG + CC	−1.19 ± 0.72	0.07–1.24	0.30	**0.09**	−1.07 ± 0.68	0.09 to 1.29	0.34	0.11	−0.15 ± 0.71	0.86–3.50	0.86	0.83

GG genotype has 0 risk allele. CG genotype has one and CC genotype have two risk allele

GG genotype is considered as a reference. Low fat, PUFA, SAFA intakes is considered as a reference. The median of RMR groups was considered for analysis as following RMR/BSA (854.50), deviation normal (−8.00), RMR/BMI (50.90), and RMR/FFM (33.73) and for RMR kg body weight was 20 kcal/24 h/kg

Crude Model: In this model, the effect of any of the confounders is not modified

Model 1: In this model, the effect of education, BMI, marriage status, age, history of weight loss in past years, energy intake, economic status, RQ and physical activity is adjusted

*p* value≤0.05

*PUFA* poly unsaturated fatty acid, *SFA* saturated fatty acid, *RMR* resting metabolic rate, *RQ* respiratory quotient, *RMR/BSA* resting metabolic rate per body surface area, *RMR/FFM* resting metabolic rate per fat free mass, *RMR/BMI* resting metabolic rate per body mass index

#### Interaction between different types of RMRs across PUFA category

**Fig. 2:**
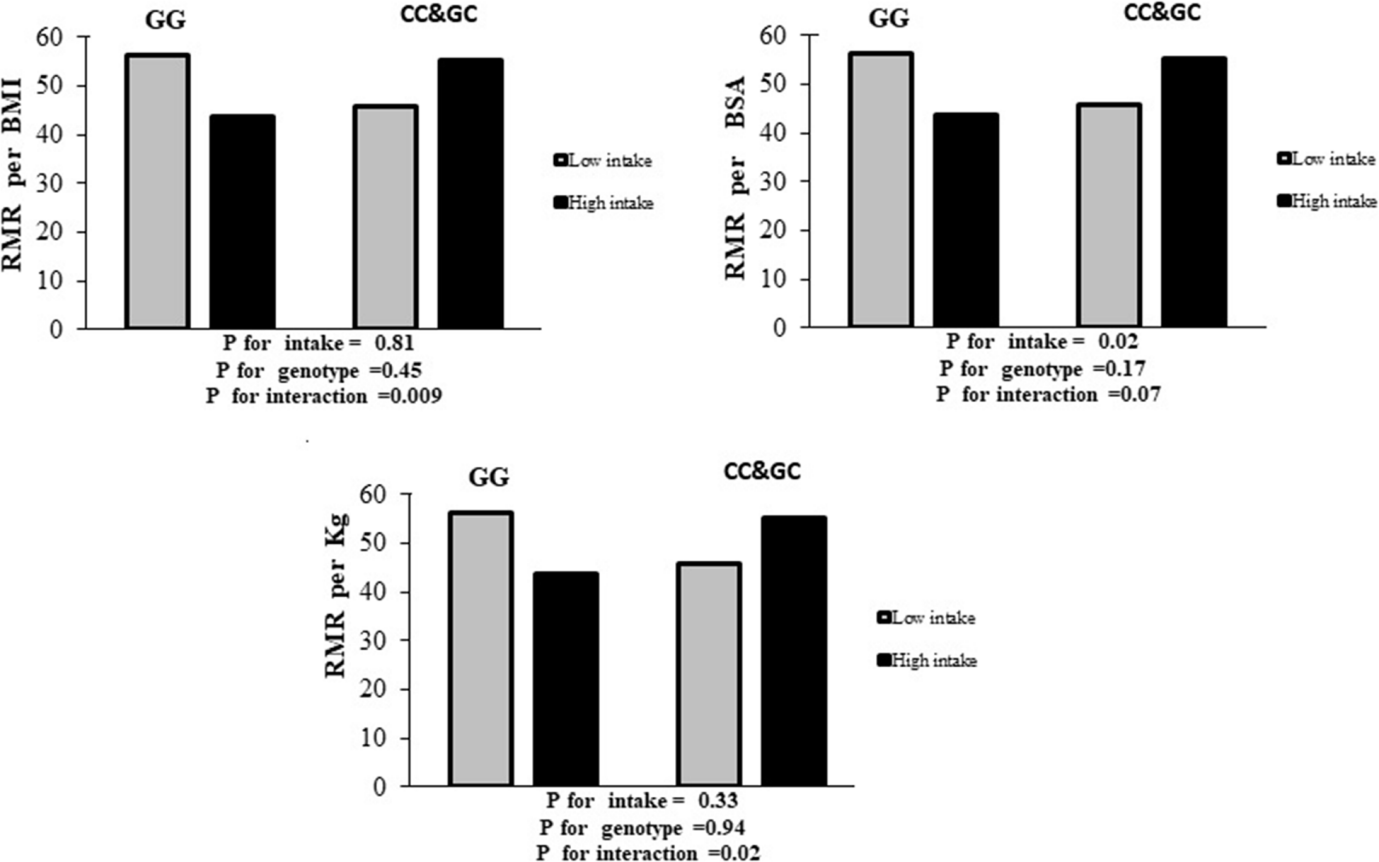
Interaction between dietary PUFA and Cry 1 genotypes on RMR disorder. Percentage of Types of RMR disorder across GC&CC and CC genotypes base on low and high dietary PUFA. A) Percentage of RMR/ kg disorder in low intake across GG and GC&CC genotypes were respiratory 56.2 and 45.7%., Percentage of RMR/kg disorder in high intake across GG, GC&CC genotypes were respiratory 43.8 and 55.3%. B) Percentage of RMR per BMI disorder in low intake across GG and GC&CC genotypes were respiratory 55.1 and 38.9%., Percentage of RMR per BMI disorder in high intake across GG and GC&CC genotypes were respiratory 44.9and 61.1%. C) Percentage of RMR per BSA disorder in low intake across GG and GC&CC genotypes were respiratory 60.2 and 45%., Percentage of RMR per BSA disorder in high intake across GG and GC&CC genotypes were respiratory 39.8and 55%. *P for interaction is for model adjusted (Potential confounders: education level, BMI, marriage status, age, history of weight loss in past year, energy intake, economic status, RQ, and physical activity)

In the crude model, there was a significant interaction between higher PUFA intake and risk allele(C) genotype group (CC + CG) in comparison with the reference group (GG) on RMR per kg body weight (β:-0.96, OR:0.38 CI:0.04–0.97; *P* = 0.04), after controlling for confounders, this association remained significant (β:-1.65, OR:0.19 CI:0.04–0.82; *P* = 0.02), such that in participants with increased intake of PUFA in the risk alleles group had 81% lower odds for higher RMR per kg compared to participants with no allele risk (GG) and a lower intake of PUFA. Also, for RMR per BSA, there was no significant association in the crude model (β: -0.94, OR:0.75 CI:0.13–1.13; *P* = 0.60), but after adjustment, there we found a significant interaction between CC + CG group with higher intake of PUFA, compared to GG group (β: -1.22, OR:0.29 CI:0.07–1.12; *P* = 0.07) (Table 4, Fig. 2), indicating that individuals in the risk allele group with higher intake of PUFA intake had 71% lower odds for a higher RMR per BSA compared to the GG group.

There was a significant interaction between PUFA intake with risk allele (C) genotype group (CC + CG) on RMR per MBI in the crude model (β: -1.38, OR:0.25 CI:0.08–0.73; *P* = 0.01), and this remained significant after adjustment for potential confounders and lead to decreased odds (β: -1.97, OR:0.13 CI:0.03–0.61; *P* = 0.00). Accordingly, this equated to an 87% reduction in the odds of higher RMR per BMI in individuals in the risk allele group (CC + CG) and with higher intake of PUFA intake, compared to participants with no allele risk (GG) and a lower intake of PUFA (Table 4, Fig. 2). We found a significant negative interaction between the CC + CG group with a higher intake of PUFA intake (β: -0.90, OR:0.40 CI:1.18–0.13; *P* = 0.09), which ameliorated after adjustment for confounding variables (*P* = 0.11). No other significant associations were found between PUFA and RMRs (Table 4).

#### Interaction between different types of RMRs across SFA categories

In the crude model, there was a significant interaction between higher SFA intake and risk allele(C) genotype group (CC + CG), in comparison with the reference group (GG), on RMR per kg (β: -1.02, OR:0.35 CI:0.13–0.97; *P* = 0.04), however, after controlling for confounders, this association was attenuated (β:-1.01, OR:0.36 CI:0.08–1.63; *P* = 0.18) (Table 4).

## Discussion

The current cross-sectional study was conducted among women with overweight of obesity to investigate the interactions between the CRY1 gene and fat intake on RMR. An important factor which can significantly influence obesity is dietary intake; however, recent research has indicated that genetic differences and variants in the human genome may alter energy expenditure and body weight [48]. Therefore, we hypothesized that individuals with the CC + GC genotypes may have lower RMR compare to individuals with GG genotypes and a high-fat diet may interact with this association. Accordingly, based on our results, Participants with risk allele(C) of rs228716 genotype group (CC + CG) and higher intake of total fat were at a 79% lower odds for higher RMR per kg body weight compared to participants with no allele risk (GG) and a lower intake of fat. Risk allele carriers with higher fat intake had 72% lower odds for higher RMR per BSA compared to no risk allele group. CC + CG group with a higher intake of total fat compared to the GG group had 72% lower odds for higher RMR per FFM. There were 70% lower odds for higher RMR deviation from normal in CC + CG group with higher intake of total fat intake, compared to the GG group. We did not detect any significant interaction between different types of RMRs across SFA and PUFA categories.

Genetic profile is an informative factor in the etiopathogenesis of obesity. In addition to gene polymorphisms, which effect on adipogenesis, there are some gene polymorphisms which can alter the regulation and level of energy balance [49]. A two year randomized weight-loss diet trial found a significant relationship between CRY2 rs11605924 and changes of RMR [50]. Moreover, in the mentioned study, it was found that dietary fat intake modified the effect of CRY2 in changes in respiratory quotient (RQ), a parameter of fuel utilization.

A 3-month low-calorie-diet interventional study among women who were at the risk of gestational diabetes revealed that G allele carriers of Cry1 rs2287161 polymorphisms presented less body weight loss and less improvement in insulin secretion, HOMA-IR, and insulin sensitivity than counterparts who were non-carriers of the G allele [51]. Indeed, previous research has shown that presence of the G risk allele of Cry1 rs2287161 polymorphisms was linked to decreased insulin secretion and sensitivity [52]. In a study consisting of African-American pregnant women, it was indicated that participants who were C allele carriers of Cry1 rs2287161 polymorphisms have lower fat intake than non-carriers [53]. Recently, Moradi et al. [54] posited that dietary fat intake may have an effect on RMR and RMR/FFM among obese women. Indeed, Moradi et al. reported that the AA genotype of PPARGC1A (rs11290186) had a positive association with PUFAs intake, even after adjustment for energy intake. Moreover, there was an interaction between total fat and SFAs intake with the PPARGC1A genotypes, and, in line with the present study, the authors found that women with a fat intake of more than 30% of calories/day had lower RMR, as well as RMR/FFM [54].

The principal mechanism of the impact of gene variants on lipid metabolism, weight changes, and RMR level is unknown. However, animal studies have demonstrated the effect of fat on expression of clock gene mRNA, lipogenic genes, and circadian balance [55, 56]. High fat intake is known to induce a decrease of the mRNA, which is needed for several different enzymes, including glutathione synthetase, superoxide dismutase, and glutathione peroxidase [57]. A high-fat diet can elicit the hyperacetylation of proteins, which is related to impaired mitochondrial function [58]. Moreover, after a high-fat diet, hyperinsulinemia and insulin resistance can occur through glucagon-like peptide-1 signaling, which is related to reducing metabolic thermogenesis and energy expenditure reduction [59].

In the present study, we did not find any significant interaction between different types of RMRs across PUFA categories. It has previously been shown that the higher intake of PUFA is beneficial for glycemic indices and lipid profile among individuals who carried G allele of ADRB2 rs1042713 polymorphism [60]. Moreover, a meta-analysis on feeding trials revealed that PUFA had some effect in improving insulin resistance [61]. Also, PUFA can, reportedly, alleviate the inflammation of adipose tissue and oxidative stress [62]. Indeed, the extant literature indicates that the composition of dietary fat is important in insulin-related processes and probably RMR. Therefore, more studies among different genders and age groups are needed to better elucidate the importance, and the manipulation, of dietary fat composition.

To the best of our knowledge, this is the first investigation on the association between GC genotypes of Cry1 rs2287161 polymorphisms, dietary fat, and the level of RMR in women with overweight of obesity. However, notwithstanding the novelty of the present study, several limitations should be considered in the interpretation of the results, including the small number of participants, considering just one gender, and the cross-sectional nature of the study. Indeed, it is, therefore, advocated that cohort studies, that include both genders, be conducted; in addition to appropriately powered sample sizes.

## Conclusion

In summary, the present study revealed that the high-fat intake, with the CC + GC genotypes, may contribute to a lower RMR in women with overweight of obesity. The present study highlights the important role of gene-diet interaction and the potential for personalized diet therapy based on genetic characteristics. Moreover, this study indicates important future research directions regarding the importance of genetic variants and their association with circadian rhythms and changes in energy expenditure. Further studies are needed to confirm the veracity our findings and to clarify the precise mechanism(s) of action.

## Data Availability

The data that support the findings of this study are available from Khadijeh Mirzaei but restrictions apply to the availability of these data, which were used under license for the current study, and so are not publicly available. Data are however available from the authors upon reasonable request and with permission of Khadijeh Mirzaei.

## Abbreviations

ANCOVA: Analysis of Covariance,
Β: Beta
BIA: Bioelectrical Impedance Analyzer
BMI: Body mass index
BSA: Body surface area
BW: Body weight
BFM: Body fat mass
CIs: Confidence intervals
Cry: Cryptochrome
CVDs: Cardiovascular diseases
EDTA: Ethylenediaminetetraacetic acid
FBS: Fasting blood sugar
FFM: Fat Free Mass
HC: Hip circumferences
HDL: High density lipoprotein cholesterol
HOMA-IR: Homeostatic model assessment insulin resistance
hs-CRP: Hypersensitive C - reactive protein
IPAQ: International Physical Activity Questionnaire
LDL: Low density lipoprotein cholesterol
RMR: Resting metabolic rate
RMR per BMI: Resting metabolic rate per body mass index
RMR per BSA: Resting metabolic rate per body surface area
RMR per FFM: Resting metabolic rate per fat free mass
RQ: Respiratory quotient
SCN: Suprachiasmatic nucleus
SDs: Standard Deviations
TC: Total cholesterol
TG: Triglyceride
WC: Waist circumference
WHR: Waist and hip
